# EIS Characterization of Ti Alloys in Relation to Alloying Additions of Ta

**DOI:** 10.3390/ma15020476

**Published:** 2022-01-08

**Authors:** Pedro P. Socorro-Perdomo, Néstor R. Florido-Suárez, Julia C. Mirza-Rosca, Mircea Vicentiu Saceleanu

**Affiliations:** 1 Mechanical Engineering Department, Las Palmas de Gran Canaria University, 35017 Las Palmas de Gran Canaria, Spain; pedro.socorro@ulpgc.es (P.P.S.-P.); nestor.florido@ulpgc.es (N.R.F.-S.); 2 Neurosurgery Department, Faculty of Medicine, “Lucian Blaga” University, 550024 Sibiu, Romania; vicentiu.saceleanu@gmail.com

**Keywords:** Ti–Ta alloys, corrosion, electrochemical impedance spectroscopy

## Abstract

The increased popularity of Ti and its alloys as important biomaterials is driven by their low modulus, greater biocompatibility, and better corrosion resistance in comparison to traditional biomaterials, such as stainless steel and Co–Cr alloys. Ti alloys are successfully used in severe stress situations, such as Ti–6Al–4V, but this alloy is related to long-term health problems and, in response, different Ti alloys composed of non-toxic and non-allergic elements such as Nb, Zr, Mo, and Ta have been developed for biomedical applications. In this context, binary alloys of titanium and tantalum have been developed and are predicted to be potential products for medical purposes. More than this, today, novel biocompatible alloys such as high entropy alloys with Ti and Ta are considered for biomedical applications and therefore it is necessary to clarify the influence of tantalum on the behavior of the alloy. In this study, various Ti–xTa alloys (with x = 5, 15, 25, and 30) were characterized using different techniques. High-resolution maps of the materials’ surfaces were generated by scanning tunneling microscopy (STM), and atom distribution maps were obtained by energy dispersive X-ray spectroscopy (EDS). A thorough output of chemical composition, and hence the crystallographic structure of the alloys, was identified by X-ray diffraction (XRD). Additionally, the electrochemical behavior of these Ti–Ta alloys was investigated by EIS in simulated body fluid at different potentials. The passive layer resistance increases with the potential due to the formation of the passive layer of TiO_2_ and Ta_2_O_5_ and then decreases due to the dissolution processes through the passive film. Within the Ti–xTa alloys, Ti–25Ta demonstrates excellent passive layer and corrosion resistance properties, so it seems to be a promising product for metallic medical devices.

## 1. Introduction

Titanium achieves its excellent corrosion protection because of the high stability of the passive layer that develops on its surface [1], which can be re-formed at body temperature and in physiological fluid if it is damaged. The increased popularity of Ti and its alloys as important biomaterials is driven by their low modulus [2,3,4], greater biocompatibility, and better corrosion resistance in comparison to traditional biomaterials, such as stainless steel and Co–Cr alloys [5].

These desirable qualities were the motivating force for the early insertion of Ti as an implantable material. Ti has low mechanical strength [6,7], and when aluminum and vanadium are incorporated in low amounts, the resistance of the alloy is greatly improved over that of titanium and the alloy could be successfully used in severe stress situations, such as Ti–6Al–4V, which has been predominantly employed.

However, Ti–6Al–4V has significant toxicity; harmful tissue reactions are caused by vanadium and the release of both V and Al ions are related to long-term health disorders such as peripheral neuropathy and Alzheimer’s and Parkinson’s diseases [8,9]. 

In response to these health problems, different Ti alloys composed of non-toxic and non-allergic elements such as Nb, Zr, Mo, Ta, etc., have been developed for biomedical applications [4,10,11,12]. However, the non-toxicity of alloying elements is only the first of the three criteria for metallic materials to be used for medical applications. The second important criterion for biomaterials is their resistance to corrosion, which also dictates the tissue compatibility and eventual osseointegration of the implant—critical aspects to consider for implant alloy design. The last important criterion is the Young’s modulus of the implant in comparation with that of the bone. A mismatch between these modules can lead to a reallocation of loads surrounding the implant, causing implant loosening [13].

In this context, binary alloys of titanium and tantalum have been developed and analyzed [14,15,16,17,18,19,20,21,22,23] and are predicted to be potential products for medical purposes; as tantalum is a non-toxic element [24], they have better compatibility with bone tissue compared with cp–Ti and Ti–6Al–4V alloys [21], and Ti–Ta alloys exhibit reduced modulus of elasticity and increased relative strength (at equivalent stiffness) compared with commercially pure titanium (cp-Ti) [25]. More than this, today novel biocompatible alloys such as high entropy alloys with Ti and Ta are considered for biomedical applications and, therefore, it is necessary to clarify the influence of tantalum in the behavior of the alloy.

Electrochemical impedance spectroscopy (EIS) is applied to characterize the behavior of different metals and alloys in various environments and to provide new information that previously could not be obtained with classical dc techniques [26,27]. Although a significant amount of research has been performed using EIS to characterize the biomaterials, little research has been conducted on EIS measurements of Ti–Ta alloys [17,28]. It is observed that it is essential for all systems to consider suitable impedance models that can be used to fit the experimental results and to provide the relevant data that characterize the corrosion process.

In this study, various Ti–xTa alloys (with x = 5, 15, 25, and 30) were characterized using different techniques. High-resolution maps of the materials´ surface were generated using scanning tunneling microscopy (STM), and information about atom distribution maps was obtained using energy dispersive X-ray spectroscopy (EDS). A thorough output of chemical composition and hence the crystallographic structure of the alloys were identified by X-ray diffraction (XRD). Additionally, the electrochemical behavior of these Ti–xTa alloys was investigated in simulated body fluid (SBF) at different potentials.

## 2. Materials and Methods

### 2.1. Material and Sample Preparation

The studied titanium tantalum alloys were Ti–5Ta, Ti–15Ta, Ti–25Ta, and Ti–30Ta from R&D CS (Research & Development Consulting and Services), Bucharest, Romania. The Ti–Ta ingots (diameter = 20 mm, length = 30 mm) were produced by levitation fusion in a high-frequency induction furnace operating with a cold copper crucible followed by a homogenization heat treatment (heating rate 5 °C/min, homogenized at 1000 °C for 8 h followed by natural cooling) in order to eliminate the segregation. The chemical composition of the alloys was determined by the supplier; for Ti and Ta content, the XRF technique was applied, while for the impurities (e.g., O, N, and C), the inert gas fusion technique was employed. The detailed chemical composition of the alloys is presented in Table 1. 

The elastic modulus E and tensile strength σ_t_ of the obtained alloys are Ti–5Ta (E = 142 GPa, σ_t_ = 381 MPa), Ti–15Ta (E = 101 GPa, σt = 402 MPa), Ti–25Ta (E = 65 GPa, σt = 464 MPa), and Ti–30Ta (E = 94 GPa, σ_t_ = 445 MPa). The experimental methods followed the ASTM E3-11 (2017) standard for metallo-graphic sample preparation [29]. The ingots were cut with minimal deformation using the Buehler IsoMet 4000 Precision Saw, (Chicago, IL, USA) and one-micron positioning allows for precise sectioning. Then, the specimens were mounted with acrylic (compression hot mounting) in order to protect edges during the polishing process. The next operation involves the grinding up to 2500 grit with SiC paper and then polishing with 0.1 µm alpha-alumina until a mirror finish is obtained in a Struers TegraPol-11 (Copenhague, Denmark) polishing machine. The samples were ultrasonically cleaned using deionized water and rinsed thoroughly with distilled water and ethanol.

### 2.2. Microstructural Characterization

EDS measurements were carried out with an environmental scanning electron microscope model FEI XL30 ESEM with an LaB6 cathode attached to an energy dispersive X-ray electron sample analyzer, model EDAX Sapphire.

The structures of the Ti–Ta alloys were investigated by high-resolution scanning tunneling microscopy (STM). All determinations were performed in air with a Hitachi TM3030 microscope that had been transversely calibrated by imaging atomically accurate oriented pyrolytic graphite. The tips were obtained by chopping a 0.20 mm Pt0.8Ir0.2 wire. The data were acquired in constant current operation with specific tunneling currents of 0.13–0.3 nA and a specimen polarization of 0.4–1.0 V. No tip-induced shifts were noted.

X-ray diffraction (XRD) determinations were made using an Empyrean diffractometer (Malvern-Panalytical). The device worked with a Cu Kα anode (1.5406 Å) in the range of 2θ = 0–64° with a step size of 0.04° at a power of 45 kV and 40 mA in Bragg–Brentano geometry. The samples have been rotated while collecting data in order to achieve better data capture. The obtained patterns were simulated in order to determine the presence of the crystalline phase, the lattice parameter, and the diameter of the grain with the assistance of Malvern-Panalytical’s HighScore Plus software.

### 2.3. Electrochemical Measurements

Electrochemical measurements were carried out with a PAR 263 A potentiostat coupled with a PAR 5210 (AMETEK, Berwyn, PA, USA) lock-in amplifier. A standard three-electrode electrochemical cell with a Pt grid as a counter electrode and a saturated calomel electrode (SCE) as a reference electrode was used. The mounted, grinded, and polished samples of Ti–xTa alloys were employed as working electrode for the electrochemical measurements. All the measurements were performed in simulated body fluid (SBF) prepared in our laboratory with the pH = 7.8 measured with a multiparameters analyzer CONSORT 831C and the composition is presented in Table 2. 

The open circuit potential was recorded for 24 h with the samples immersed in SBF solution. The potentiodynamic polarization curves were obtained with a scanning rate of 0.166 mV/s in the potential range −800 to +2000 mV vs. SCE. The polarization resistance R_p_ was calculated from traces of the polarization curve at ±10 mV versus open circuit potential.

The effect of the potential on the passive film of Ti–Ta alloys operating in a simulated physiological environment was evaluated by electrochemical impedance spectroscopy (EIS). The AC potential amplitude was set at 10 mV, and single sine wave recordings were performed at frequencies in the range of 10^−1^ and 10^5^ Hz for all specimens. To characterize the oxide layer, impedance spectra were registered in the range of −400 to 2000 mV with a step of 100 mV by continuously polarizing the electrodes and letting the system equilibrate for 600 s at every potential. For the numerical fit of the measured impedance data, the software program ZSimWin was used.

All the electrochemical tests were normally repeated three or four times to ensure that they presented reasonable reproducibility.

## 3. Results and Discussions

**Figure 1:**
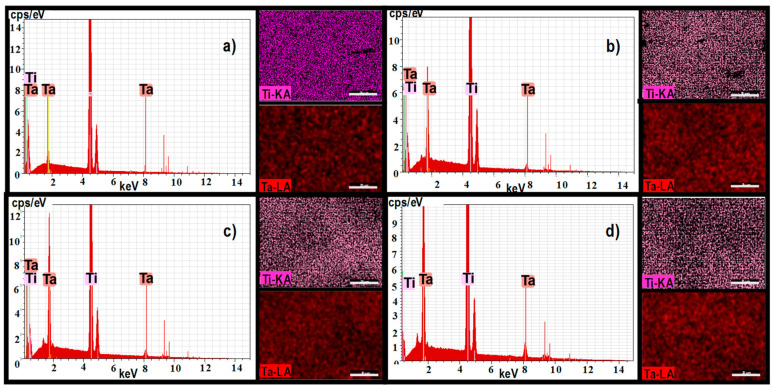
Atoms distribution of Ti and Ta in the studied alloys: (**a**) Ti5Ta, (**b**) Ti15Ta, (**c**) Ti25Ta, and (**d**) Ti30Ta.

The EDS analysis has been performed on micro-zones of the same square area, and the elemental distribution maps (see Figure 1) and chemical composition for the four alloys (see Table 3) are presented. It can be observed that there are no impurities in the metallic mass and titanium and tantalum were the only identified elements.

**Figure 2:**
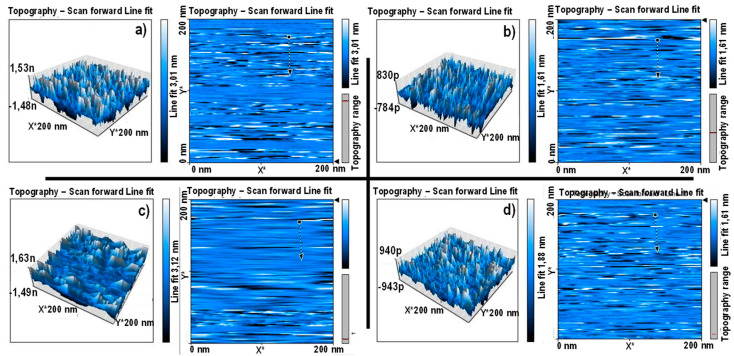
Representative scanning tunneling microscopy (STM) images of surface topographies for (**a**) Ti5Ta, (**b**) Ti15Ta, (**c**) Ti25Ta, and (**d**) Ti30Ta.

In Figure 2, we can observe the surface topography acquired by scanning tunneling microscopy (STM). A three-dimensional analysis of the surface topography reveals nanoscale deviations within an overall homogeneous nanoscale architecture found on the surface of the Ti–Ta alloy.

Nanoscale deviations, suggestive of the topography present in natural tissue, have been repetitively demonstrated to enhance the protein-specific adsorption, thus further driving cellular activity and cell–cell relationships [30]. Thus, these features enable a very promising interface to enhance cellular interaction because of increased nanoscale roughness of the material interface.

Pure titanium has a closed hexagonal structure (HCP), i.e., an α-phase at room temperature. At temperatures above 883 °C, there is a body-centered cubic (BCC) structure, i.e., a β-phase. The β-phase is stable at temperatures below 883 °C with the addition of β-stabilizers, and its stability depends on the amount of β-alloying elements. The quantity of β-stabilizer required to retain the pure β-phase at room temperature depends on the molybdenum equivalence, a rule derived from the analysis of binary titanium alloys. Molybdenum equivalence is given by [31]:

Mo_eq_ = 1.0 Mo + 0.67 V + 0.44 W + 0.28 Nb + 0.22 Ta + 1.6 Cr +……−1.0 Al

**Figure 3:**
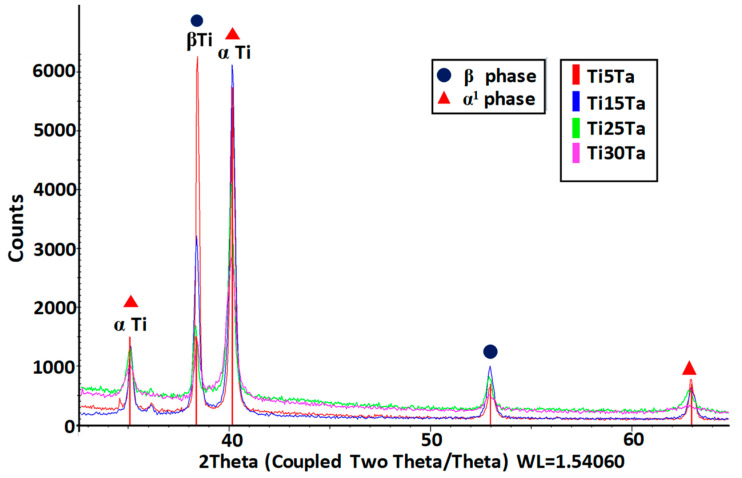
XRD pattern for TixTa alloys.

In general, a molybdenum equivalency of approximately 10 is necessary to stabilize the β-phase while quenching [14], and the critical value to reach a complete β-phase is ap-proximately 25. As we can see for the studied Ti–Ta alloys, the Moeq is below the mini-mum value needed for a fully stable β phase, and the microstructure is made up of α″ grains within β grains. Figure 3 presents the XRD patterns taken from the Ti–xTa alloys. 

Because the Ta element is a β phase former in the Ti-based alloys and Moeq is low, the microstructures of all studied samples are clearly a mixture of α´´ phase (orthorhombic structure) and β phase. However, the intensity of α´´phase decreased as the concentration of Ta increased, and this is attributed to the variation of the volume fraction of α´´ phase in the matrix.

### 3.1. Electrochemical Impedance Spectroscopy

**Figure 4:**
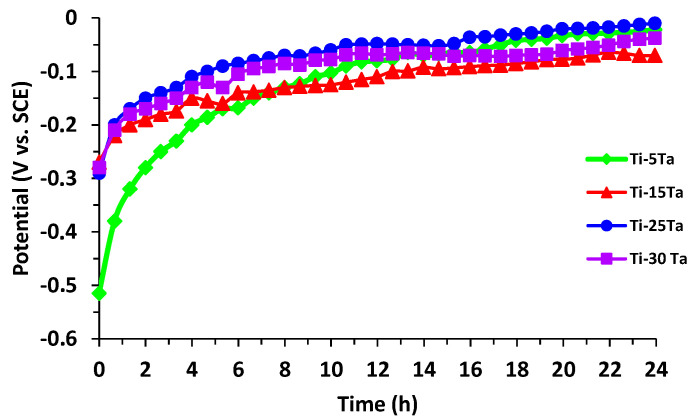
Open circuit potential curves for Ti–xTa alloys during 24 h immersion in SBF.

**Figure 5:**
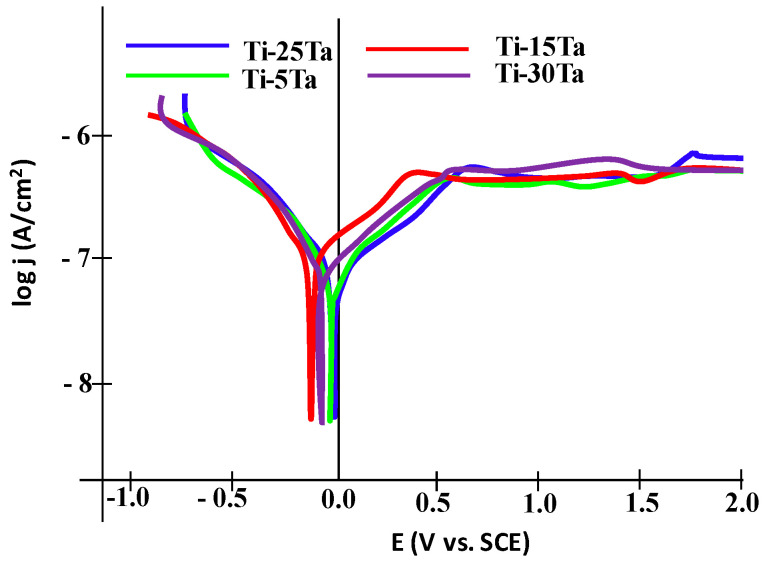
Polarization curves for Ti–xTa alloys.

Figure 4 shows the open circuit potential curves for all four Ti–xTa alloys immersed for 24 h in simulated body fluid. It can be observed that following the immersion, an abrupt displacement of the potential has taken place towards positive values during a pe-riod of 2–6 h. Afterwards, the open circuit potential continued to increase slowly, suggesting the growth of a passive layer on the metallic surface. The linear polarization curves, in semilogarithmic coordinates for the tested Ti–xTa alloys in simulated body fluid, are displayed in Figure 5, and Table 4 shows the instantaneous corrosion parameters in this physiological environment. All samples are characterized by high values of the polarization resistance R_p_ (10^5^ Ω·cm^2^).

The electric potential difference between the reference electrode and metal interface is a relevant factor directly related to the surface conditions. EIS tests have been carried out at various potentials in three areas: cathodic–anodic transition, passive transition, and quasi-transpassive transition. The impedance results will be used to compare the effect of the potential on the properties of the passive layer.

#### 3.1.1. Plots Interpretation

**Figure 6:**
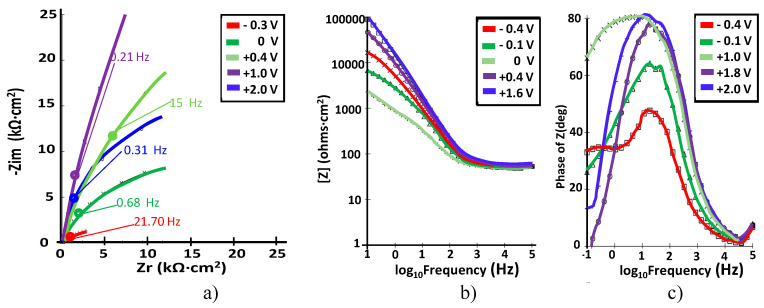
(**a**) Nyquist; (**b**) Bode –IZI and (**c**) Bode-phase spectra at different potentials for Ti–5Ta in simulated body fluid at pH = 7.8.

**Figure 7:**
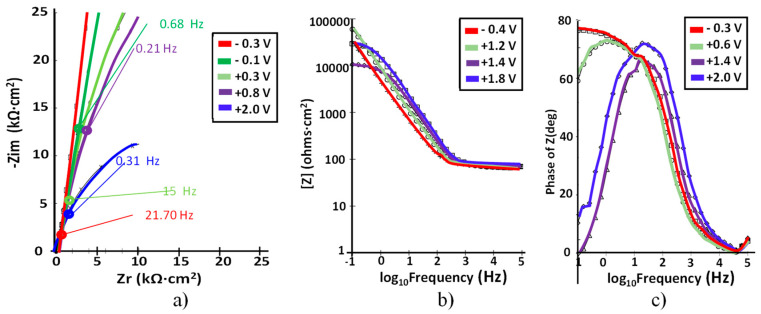
(**a**) Nyquist; (**b**) Bode –IZI and (**c**) Bode-phase spectra at different potentials for Ti–15Ta in simulated body fluid at pH = 7.8.

**Figure 8:**
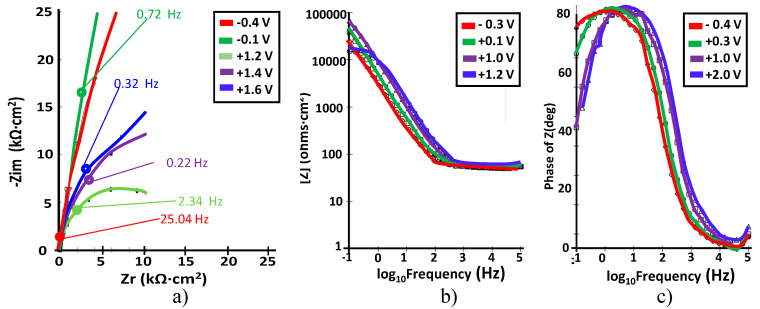
(**a**) Nyquist; (**b**) Bode –IZI and (**c**) Bode-phase spectra at different potentials for Ti–25Ta in simulated body fluid at pH = 7.8.

**Figure 9:**
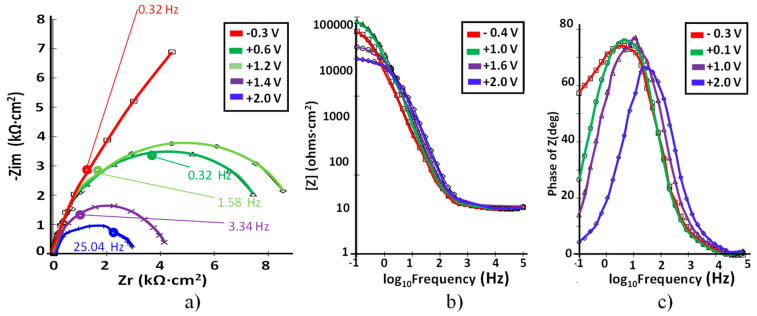
(**a**) Nyquist; (**b**) Bode –IZI and (**c**) Bode-phase spectra at different potentials for Ti–30Ta in simulated body fluid at pH = 7.8.

The Nyquist plots correspond to the impedance of Ti–5Ta, Ti–15Ta, Ti–25Ta, and Ti–30Ta at different potential values. Even the EIS data were recorded within the −0.4 V ≤ E ≤ 2.0 V potential range with a step of 100 mV; not all the obtained curves are shown in Figure 6, Figure 7, Figure 8 and Figure 9.

#### 3.1.2. Ti–5Ta

The corresponding EIS plots are presented in Figure 6.

At all potentials, a near capacitive response was detected, characterized in Nyquist plots (see Figure 6a) by incomplete semicircles. In the higher frequency band (1–100 kHz), the Bode plot (Figure 6b) shows constant values (horizontal line) of log |Z| versus log(f) with a phase angle approaching 0°. From 0 to 1 V, in the wide range of low and medium frequencies, the spectra show a linear slope of approximately –1 in log |Z| as the frequency decreases, while the phase angle values are close to 80°. This is the typical response of a compact passive film capacitor. The acquired values for passive film resistance are high until 1.8 V, after which they decrease with the potential. In Figure 6c, it can be observed that the phase angle observed for Ti–5Ta was encountered in the range of about −65° to −80°, suggesting a highly stable film on Ti–5Ta [19]. At a potential value higher than 0 V, a unique peak is noted in the phase angle graphs, which indicates the engagement of one relaxation time.

#### 3.1.3. Ti–15Ta

The corresponding EIS plots are presented in Figure 7.

For this alloy, the shape of the impedance data is similar to that of Ti–5Ta but the response of the compact passive oxide can be observed until 1.2 V, with no modifications of the value of the electrolytic solution resistance (see Figure 7b). Bode-|Z| spectra displayed a linear slope of about −1 and high impedance values (order of 10^5^ Ω·cm^2^) in the low and middle frequency ranges, which represent the characteristic response of a capacitive behavior of the passive film [32]; after a potential value of 1.2 V, the impedance values slightly decreased over time as result of the low dissolution of the passive layer [10].

#### 3.1.4. Ti–25Ta

The corresponding EIS plots are presented in Figure 8.

For this alloy, we can also observe a strong change in the shapes of the impedance data, but at lower potential than for Ti–5Ta and Ti–15Ta. This change takes place between 0.8 and 1.0 V. The Nyquist plots illustrate, at all applied potentials, one depressed semicircle, suggesting non-ideal capacitive behavior (high corrosion resistance) for Ti–25Ta. When the semicircles deformed and their diameters decreased over time, it shows some dissolution processes through the passive film. During the potential scan, the electrolytic resistance is constant (60 Ω·cm^2^), and the passive layer capacitance slowly decreases with the potential. With the Ti–25Ta alloy, the best results in EIS spectra were obtained, completing the lowest elastic modulus and the highest ratio of strength to modulus among Ti–Ta alloys [33]. It can be observed that Bode-|Z| impedance plots showed linear portions with the slope amounting to −1 (from −0.96 to −0.99) over a large frequency range (from 100 mHz to 100 kHz). At all analyzed potential values, a single peak is observed in phase angle plots, which indicates the involvement of one relaxation time.

#### 3.1.5. Ti–30Ta

The corresponding EIS plots are presented in Figure 9.

It can be observed that the Nyquist plots revealed the same capacitive loops but with the lowest impedance values compared with the other alloys. It can be seen that the Bode-|Z| impedance plots exhibited linear parts with a slope around −1 only at intermediate frequencies. It was found that the maximum phase angle observed for Ti–30Ta was the lowest among all the values obtained for the Ti–Ta alloys. It can be observed that the electrolytic resistance was constant during the experiments (27 Ω·cm^2^).

### 3.2. Equivalent Circuits

**Figure 10:**
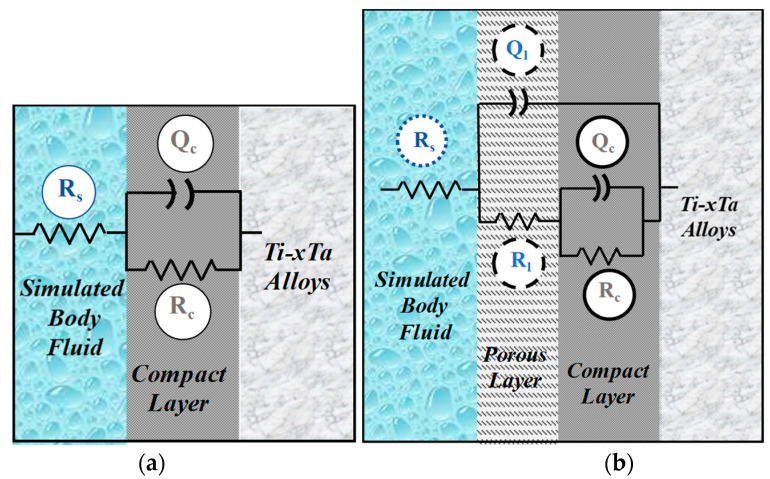
Equivalent circuits used for fitting the experimental data with: (**a**) one time-constant; (**b**) two time-constants.

The obtained impedance data were matched by employing a nonlinear regression approach using the Randles equivalent circuit (see Figure 10a) [34,35,36]. This widely used electrical equivalent circuit involves an ohmic solution resistance (R_s_) in series with the parallel arrangement of a CPE (Q_l_) and a resistor (R_l_). R_l_ is a numerical indicator that provides a value for the corrosion resistance of the passive layer that acts as a damper to the flux of electrons (resistance) across the metallic material/electrolyte interface and gives a measure of the level of protection provided by the passive layer that forms at the inter-face [10,37]. The employment of a constant phase element (Q_l_) in place of an “ideal” capacitor was required due to the inhomogeneous nature of the passive layer formed on the alloy surface and because of the differences between the flat surface of an ideal capacitor. From the obtained data, it was found that the value of R_s_ does not change significantly during the test due to the fact that the controlling factors that affect its magnitude, as the stability of the exhibited surface and the number of ions in the simulated body fluid solution do not change.

In the corrosion process, a passive layer is developed on the metallic surface and the charge transfer reaction that occurs can be disregarded and the resulting measured impedance could be assigned to the impedance of the passive film. The components of the equivalent circuit are:

R_s_—ohmic resistance of the physiological solution

R_l_—resistance of the passive layer

C_l_—capacitance of the passive layer

The analysis of the impedance plots was carried out by fitting these data with ZSimpWin software. The performance of the fit to the equivalent circuit was assessed in the first place by the chi-square value and in the second place by comparing experimental data with simulated data.

In place of capacitance, a constant phase element (CPE), representing the shift from the real capacitive performance, was employed. The impedance of a CPE is characterized by [27]:

(1) $$Z_{\mathrm{CPE}}=\frac{1}{Y^{0}{\left(j\omega \right)}^{n}}$$

where:

− Z is the impedance of the constant phase element CPE
− j is the imaginary number (j^2^ = −1)
− ω is the angular frequency (rad·s^−1^)
− nπ/2 is the constant phase angle of the constant phase element (rad)
− Y^0^ is the constant of the constant phase element [S(s·rad^−1^)^n^]

According to all Bode plots and our previous work [10], only one time constant can be clearly distinguished; however, at low frequencies, no clear definition of the second time constant can be observed so only the high–medium frequencies’ data have been analyzed in this work and only the simple circuit was used. Different studies [38,39] have proven that data at low potentials have indicated the growth of a single layer of oxide on titanium characterized by one-time constant equivalent circuit; the same circuit was reported by others who studied the corrosion of tantalum either in its pure form or as a coating [40,41,42,43].

When the impedance spectra were fitted to the equivalent circuits presented in Figure 10, a CPE element was used because, generally, a distributed relaxation feature is presented for TiO_2_ films.

In order to obtain the total impedance of the equivalent circuit, we determine the admittance of the parallel arrangement (R**_l_**Q**_l_**):

(2) $$\frac{1}{Z_{l}}=\frac{1}{Z_{R_{l}}}+\frac{1}{Z_{Q_{l}}}$$

Because the roughness factor “n” is > 0.85, near to 1, the resulting Y^0^ value of Q_l_ is assumed to be C_l_ in the following discussion:

(3) $$\frac{1}{Z_{l}}=\frac{1}{R_{l}}+{j\;w\;C}_{l}$$

**Figure 11:**
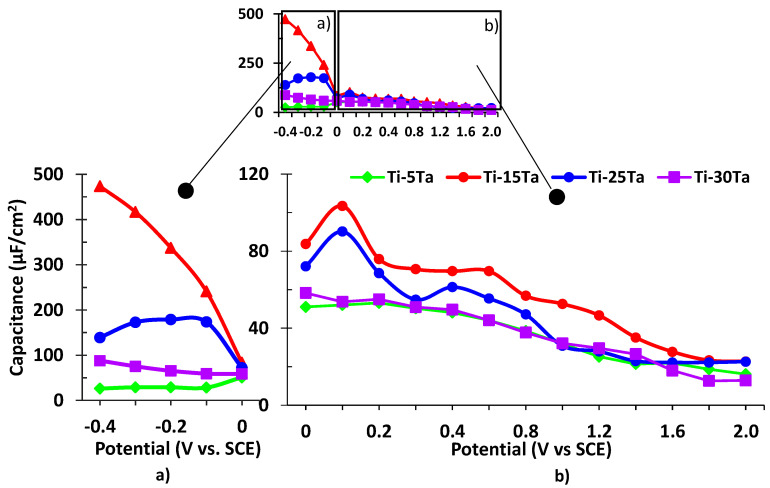
Capacitance of the passive films for Ti–Ta alloys in simulated body fluid: (**a**) at negative potentials; (**b**) at positive potentials.

**Figure 12:**
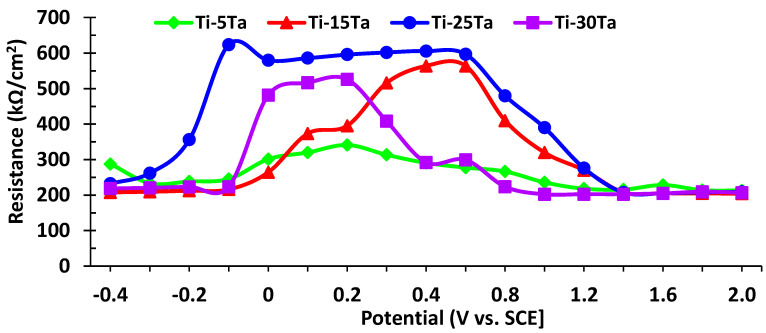
Resistance of the passive films for Ti–Ta alloys in Ringer’s solution.

The diagnostic considerations for the selection of equivalent circuits for modeling the impedance data can be resumed by visual observations of the shifts in the experimental Bode diagrams with the change of potential and concentration of the alloying metal. It appears that, in the passive zone potentials (mostly strictly capacitive impedance), the Bode graphs provide a good fit if the total impedance is modeled following the circuit in Figure 10a. The obtained results of C_l_ and R_l_ are presented in Figure 11 and Figure 12, respectively.

Figure 11 points to the fast decreasing of the passive film capacitance. A high initial C_l_ is compatible with a high initial active surface and a high roughness factor. The evolution of C_l_ with the potential is also compatible with the decreasing of the active surface due to the increasing of the passive layer thickness, with all the alloys eventually reaching the same order of film thickness.

In Figure 12, the resistance of the passive film (kΩ·cm^−2^) as a function of potential is presented. It can be observed that the value obtained for Ti–25Ta is higher than that of the other alloys, which depicts its highest corrosion resistance in simulated body fluid. This is due to the chemical composition of this alloy, which is closed to the compositional boundary between the α´ and α´´ phase and has the lowest elastic modulus and the highest ratio of strength to modulus among Ti–Ta alloys [9].

The shape of the potentiodynamic polarization curves indicated that all the Ti–xTa alloys are passivating immediately at immersion in simulated body fluid, a behavior that can be termed stable passivity. In these conditions, after 24 h of immersion, the passive film is thicker and the EIS data can be fitted with good results (chi^2^ of 10^−4^ order) by the two-time equivalent electrical circuit presented in Figure 10b. In Table 5, the main parameters of the proposed circuit for all the studied alloys are presented. Polarization resistance R_p_ is represented by the sum of the resistance of the porous passive film and the compact barrier layer (R_l_ + R_c_) [44]. The value of the “n” parameter corresponds to the extent of dispersion and is attributed to the surface inhomogeneity. The values of n_c_ are almost 1, and Q_c_ is similar with a pure capacitor. The values of n_p_ are lower than those of n_c_, and this may be due to the higher roughness and diffusion through the outer layer. The simulated data are in good agreement with the experimental data, and chi-square values of 10^−4^ were obtained. 

The corrosion resistance of an alloy could be characterized by the polarization resistance at the corrosion potential. This parameter can be calculated in two ways: (1) taking the slope of the E vs. I relationship in the range of ± 10 mV vs. open circuit potential; (2) from the fitting results of EIS spectra with all the implicated resistances [45]. Comparing the values obtained by the two techniques, it is observed that, for low concentrations of tantalum (Ti–5Ta and Ti–15Ta), the values of corrosion resistance obtained by EIS are lower than those of the DC experiments. This is probably due to the fact that with the shift of the potential towards more positive values vs Ecorr, titanium forms a more compact oxide layer than it does at the corrosion potential. At higher Ta concentrations (Ti–25Ta and Ti–30Ta), Ta_2_O_5_ plays a more important role in the compactness of the passive film and the corrosion resistance values from EIS and polarization curves are approximately the same. It can be observed that the highest corrosion resistance of Ti–xTa alloys was obtained for Ti–25Ta.

Titanium is not a noble metal, and if this metal generally exerts a high electrode potential, it is because a passivating film of oxide is formed on its surface; the stability of this layer protects the metal from further deterioration.

During the anodic polarization, a film of TiO_2_ is formed:

(4) $$\mathrm{Ti}+2H_{2}O\;\to {\mathrm{TiO}}_{2}+4H^{+}+4e^{-}$$

For Ti, the Porbaix diagram of Ti–H_2_O indicates that the substantial decrease in the passive film resistance at potentials close to 1 V can be attributed to the development of TiO_3_–H_2_O on the surface of the alloys.

The Pourbaix Ta–H_2_O diagram [46] shows that the high strength of tantalum is attributed to the development of a protective film of tantalum pentoxide (Ta_2_O_5_). Tantalum, during the anodic polarization, tends to cover itself with this layer of Ta_2_O_5_, which has good qualities (compactness, continuity, etc.). The reaction that takes place is:

(5) $$2\mathrm{Ta}+5H_{2}O\;\to {\mathrm{Ta}}_{2}O_{5}+10H^{+}+10e^{-}$$

Tantalum pentoxide can exist in the hydrated state; it has a variable percentage of water of crystallization and is attributed the formula Ta_2_O_5_·H_2_O or HTaO_3_.

## 4. Conclusions

1. The Nyquist plots for all the Ti–Ta alloys show the same incomplete semicircles with large diameters increasing with the potential (up until a critical value for each alloy) due to the improvement of the protective properties of the passive film formed on the surface of the alloy.
2. For all Ti–Ta alloys, the Bode phase plots exhibited one phase angle—typical for a capacitive barrier passive layer formed on the surface of an alloy.
3. Impedance spectra are fitted with a one-time constant equivalent circuit, common for a compact oxide layer, for all Ti–Ta alloys in extra-cellular fluids. After a long immersion period in simulated body fluid, the passive film is thicker and develops a bi-layer structure: an outer porous layer and an inner compact layer. Tantalum addition increases the stability of the passive film due to the development of Ta_2_O_5._
4. Of the Ti–Ta alloys, Ti–25Ta demonstrates excellent passive layer and corrosion resistance properties, and thus it seems to be a promising product for metallic medical devices.

## Figures and Tables

**Table 1:** Chemical composition of Ti–xTa alloys.

Alloy	Ti	Ta	O	N	C
	(wt. %)	(wt. %)	(wt. ppm)	(wt. ppm)	(wt. ppm)
Ti–5Ta	94.20 ± 0.06	4.92 ± 0.05	180 ± 14	80 ± 7	110 ± 8
Ti–15Ta	84.41 ± 0.06	14.82 ± 0.07	162 ± 11	75 ± 5	101 ± 6
Ti–25Ta	74.52 ± 0.05	24.89 ± 0.09	158 ± 12	82 ± 6	103 ± 5
Ti–30Ta	69.61 ± 0.07	29.67 ± 0.05	172 ± 11	78 ± 5	111 ± 4

**Table 2:** Composition of simulated body fluid (SBF).

Compound	Composition [g/L]
NaCl	6.80
KCl	0.40
CaCl_2_	0.20
MgSO_4_·7H_2_O	0.20
NaH_2_PO_4_·H_2_O	0.14
NaHCO_3_	2.20
Glucose	1.00

**Table 3:** EDS global analysis on micro-areas for Ti–xTa alloys.

Alloy	Ti			Ta		
	at.%	wt.%	Error, %	at.%	wt.%	Error, %
Ti–5Ta	99.01	94.95	1.22	0.88	4.38	3.67
Ti–15Ta	95.62	83.95	1.95	4.38	14.87	2.24
Ti–25Ta	91.76	74.63	2.03	8.23	24.83	2.15
Ti–30Ta	90.06	69.74	1.81	9.95	29.72	1.98

**Table 4:** Corrosion parameters from polarization curves (mean ± SD).

Alloy	E_corr_	i_corr_	i_pass_	R_p_
	(mV vs. SCE)	(µA/cm^2^)	(µA/cm^2^)	(kΩ·cm^2^)
Ti–5Ta	−22 ± 4	0.67 ± 0.06	0.61 ± 0.11	522 ± 16
Ti–15Ta	−70 ± 3	0.52 ± 0.12	0.58 ± 0.05	501 ± 23
Ti–25Ta	−10 ± 3	0.43 ± 0.04	0.51 ± 0.16	598 ± 28
Ti–30Ta	−38 ± 4	0.58 ± 0.21	0.57 ± 0.18	495 ± 12

**Table 5:** Circuit parameters calculated from the fitting of the EIS spectra at corrosion potential to the equivalent circuit from Figure 10b.

Alloy	R_s_ (Ω·cm^2^)	Y^0^_p_·10^5^ (S·s^n^/cm^2^) 0 < n < 1	n_o_	R_l_ (Ω·cm^2^)	Y^0^_c_·10^5^ (S·s^n^/cm^2^) 0 < n < 1	n_c_	R_c_ (kΩ·cm^2^)	χ^2^·10^4^
Ti–5Ta	34.2	1.72	0.59	500.6	2.71	0.89	153.1	7.24
Ti–15Ta	59.0	9.90	0.78	887.5	17.6	0.91	322.4	4.37
Ti–25Ta	11.5	1.74	0.72	14.5	4.31	0.94	577.3	4.89
Ti–30Ta	27.1	6.13	0.79	28.1	1.10	0.90	485.9	1.88
